# Circulating MicroRNA Profiles Differ between Hyperglycemia and Euglycemia in Coronary Heart Disease Patients

**DOI:** 10.1155/2017/9192575

**Published:** 2017-10-30

**Authors:** Yunyao Jiang, Nan Liu, Bingjie Xue, Jincai Hou, Chengren Lin, Jianxun Ren, Jianxun Liu

**Affiliations:** ^1^Institute of Basic Medical Sciences, Xiyuan Hospital, China Academy of Chinese Medical Sciences, Beijing 100091, China; ^2^Beijing Key Laboratory of TCM Pharmacology, Xiyuan Hospital, China Academy of Chinese Medical Sciences, Beijing 100091, China; ^3^Jing-Jin-Ji Joint Innovation Pharmaceutical (Beijing) Co., Ltd., Beijing 100083, China; ^4^Beijing University of Chinese Medicine, Beijing 10029, China

## Abstract

Coronary heart disease (CHD) has become one of the leading causes of death and functional impairment in the world. Hyperglycemia is associated with an increased risk of cardiovascular disease. It was speculated that miRNAs in peripheral blood were a primary parameter in discriminating CHD. The biological characteristics of coronary heart disease with hyperglycemia (HCHD) and coronary heart disease with euglycemia (ECHD) were investigated in the study. Circulating miRNAs from 26 HCHD patients and 42 ECHD patients were identified by microarrays. Compared with the healthy patients, 15 and 20 differentially expressed miRNAs were identified in HCHD and ECHD groups, respectively. Gene ontology analysis was carried out by DAVID and functional annotations of the miRNA targets related to ATP binding, cellular components, protein binding, RNA binding, DNA binding, and so on. KEGG database was used for pathway analysis. Eleven pathways were identified in both HCHD and ECHD groups. Furthermore, 13 and 3 pathways were only identified in HCHD or ECHD group, respectively. And then, miRNA-gene regulatory networks were constructed to study the relationship between differentially expressed miRNAs and genes. This suggested that hsa-let-7c-5p and hsa-miR-24-3p might have the most important function for hyperglycemia in coronary heart disease patients.

## 1. Introduction

Coronary heart disease (CHD) is caused by atherosclerosis and thrombosis or atherothrombosis and has become one of the leading causes of death and functional impairment in the world [1, 2]. It is reported that CHD may kill approximately seven million people and result in 129 million losses of disability-adjusted life years (DALYs) annually [3]. Studies reveal that more than 20% of patients with CHD have diabetes, about 35% are obese, about 45% have metabolic syndrome, more than 50% have hypertension, and almost 60% have dyslipidemia [4]. Hyperglycemia, not only in diabetic but also in the nondiabetic range, is associated with an increased risk of cardiovascular disease. This has been demonstrated by several trials and evidences that glycemic control appears to be linked to a reduced coronary artery disease [5]. Several mechanisms that hyperglycemia exacerbate myocardial damage and worsen the prognosis in patients with acute coronary syndrome have been revealed. Hyperglycemia results in myocardial cellular injury by producing free radicals and then inducing oxidative stress. In addition, hyperglycemia can promote osmotic diuresis and lower the circulating volume to affect cardiac contractility and decrease both end-diastolic volume and stroke volume [6].

MicroRNAs (miRNAs) are short endogenous and single-stranded noncoding RNA molecules that bind to the 3′UTR of their target messenger RNA (mRNA) and inhibit or decrease translation of the mRNA into protein to regulate target gene expression [7, 8]. MiRNAs regulate approximately 60% of human protein-coding genes and perform their functions in a number of biological processes such as cell development, differentiation, metabolism, immunity, apoptosis, and proliferation, which is conducive to many physiological and pathological conditions including cardiovascular diseases [9, 10]. Atherosclerosis of the coronary arteries is a very complex process and is responsible for the development and clinical manifestations of CHD. The role of miRNAs in the process of manifestations has been evaluated through many researches and it is now recognized that miRNAs are connected to nearly all steps of atherogenesis, including beneficial and detrimental effects such as endothelial damage and dysfunction, monocyte-wall invasion and activation, lipoprotein formation, and platelet and vascular smooth muscle cell function exerting [11]. MiRNAs can give rise to degradation or blockade of protein translation because of negative regulation to the gene expression by binding to target mRNAs. MiRNAs can bind to target mRNAs to regulate the gene expression and lead to degradation or blockade of protein translation. In the circulation, they are resistant to degradation by being packaged as microvesicles, bound to protein complexes, or bound to high-density lipoproteins [12]. Therefore, miRNAs can be easily measured in circulation and the levels of miRNAs have been used as potential blood biomarkers for various pathologies. MiRNAs have been used as biomarkers in cancer diagnosis by detecting circulating levels of specific miRNAs, which have been associated with the pathogenesis of obesity, diabetes mellitus, and coronary artery disease and used in the diagnosis of myocardial infarction [13].

The circulating miRNAs are believed to come from circulating tumor cells and blood cells or other tissue cells affected by disease also directly release miRNAs into the blood stream [14]. Turchinovich et al. reported the theory of cell-cell communication via extracellular miRNA [15]. And they described the evidence that the short distance communication of cells was realized with extracellular miRNA. The long-range communication between different intercellular sites is also realized through the current models for the functionality of circulating miRNAs [16].

It was speculated that miRNAs in peripheral blood were a primary parameter in discriminating CHD. The biological characteristics of CHD with hyperglycemia (HCHD) and CHD with euglycemia (ECHD) were researched by investigating circulating miRNA profiles and bioinformatics of HCHD and ECHD patients. And the common or different mechanisms between HCHD and ECHD were analyzed by gene ontology (GO) and pathway analysis of miRNA targets. Functional annotations of the miRNA targets are related to transcription, cellular components, protein binding, RNA binding, and DNA binding, and so on. Twelve pathways, including p53 signaling pathway, cell cycle, FoxO signaling pathway, viral carcinogenesis, and chronic myeloid leukemia, were identified in both HCHD and ECHD groups. Furthermore, 13 and 3 pathways were only identified in HCHD or ECHD group, respectively.

## 2. Materials and Methods

### 2.1. Participant Recruitment

Ethical approval for this study was obtained and all procedures in this study were approved by the Medical Ethical Committee of Xiyuan Hospital of China Academy of Chinese Medical Sciences. A total of 87 volunteers were recruited from Xiyuan Hospital of China Academy of Chinese Medical Sciences. Among them, 19 healthy volunteers (HV) reported no CHD and exhibited a normal syndrome as judged by doctors. Of the 68 patients, 26 fulfilled study inclusion criteria for HCHD group and 42 fulfilled study inclusion criteria for ECHD group. The clinical studies followed the Helsinki declaration guidelines. Written informed consent was obtained from all volunteers.

### 2.2. Serum Sample Collection and Handling

Venous blood was obtained from each volunteer. Ten milliliters of venous blood was collected from ulnar vein and transferred into a vacuum blood collection tube. Samples were placed immediately in ice for blood coagulation and were centrifuged at 3000 rpm for 20 min at 4°C to obtain serum. The obtained serum was taken to the study laboratory within 10 minutes.

### 2.3. RNA Isolation and Amplification

Total RNA including miRNA was isolated from the serum samples using Trizol reagent (Invitrogen, Canada) and purified using RNeasy Mini Kit (Qiagen, German) according to the protocol provided by manufacturers. It is ensured that slow molecular weight RNA can be retained in the purification. RNA concentration was determined by measuring the absorbance at 260 nm and quality control standard was* A*(absorbance)_260_/*A*_280_ = 1.8–2.1, using NanoDrop 2000 spectrophotometer (Thermo Fisher Scientific, USA).

### 2.4. Affymetrix miRNA Microarray

RNA was labeled with FlashTag Biotin HSR RNA labeling kit according to the manufacturer's instructions (Genisphere, USA). The Labeled RNAs were then hybridized to the Affymetrix GeneChip miRNA 3.0 arrays on the basis of the user manuals. Affymetrix Expression Console Software (version 1.3.1) was used to analyze the arrays, including data normalization, summarization, and quality control assessment. Median-centric normalization was used for the custom miRNA oligonucleotide chips and Affymetrix chips were normalized with the robust multichip analysis (RMA) procedure. Differentially expressed miRNAs were identified based on RVM *t*-test analysis. *p* values were adjusted and corrected according to the Benjamini-Hochberg procedure and FDR (false discovery rate) < 0.05 was chosen as the cutoff criteria. Differentially expressed miRNAs were considered to be upregulated or downregulated with at least 1.5-fold change in either direction with FDR (adjusted *p* value) < 0.05.

### 2.5. Target Prediction of miRNA and Bioinformatic Analysis

The potential human miRNA targets were predicted with Tarbase (version 7.0) [17] and miRTarBase 2016 [18]. And the intersection set of these 2 databases was used for bioinformatic analysis. In order to investigate the biological process, cellular component, and molecular function of differentially expressed miRNA targets, GO analysis was carried out using DAVID [19] Bioinformatics Resources 6.8 databases. Functional categories were enriched within genes (FDR < 0.05) and the top 10 GO functional categories were selected. DAVID that assigned Kyoto Encyclopedia of Genes and Genomes (KEGG) database was used for pathway analysis. The pathway analysis may discover a relation that was not easily visible from the changes of individual genes. The involvement of coexpressed genes in biological pathways was determined by pathway analysis. Pathways that had significant changes of FDR < 0.05 were identified for further analysis. And the genes that significantly regulated pathways were selected for miRNA-gene regulatory network analysis. MiRNA-gene regulatory networks were constructed to study the relationship between differentially expressed miRNAs and genes in the context of biological pathways [20]. The miRNA-gene regulatory networks were constructed by Cytoscape 3.5.1 software [21] and betweenness centrality was used for topological analysis.

## 3. Results

### 3.1. Identification of miRNAs

**Figure 1:**
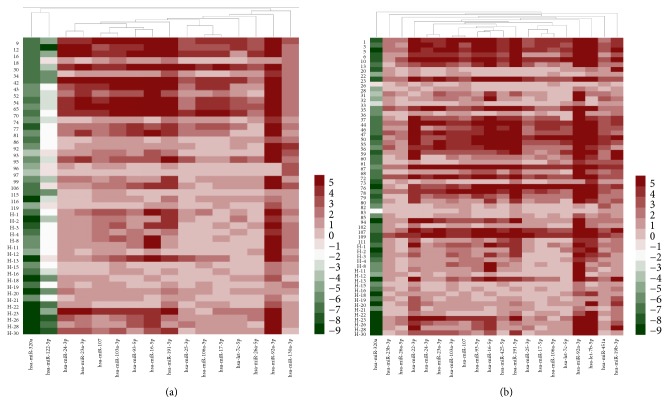
Cluster analysis of the differentially expressed microRNAs in the plasma compared between the HCHD patients and the healthy volunteers (a) or the ECHD patients and the healthy volunteers (b).

**Figure 2:**
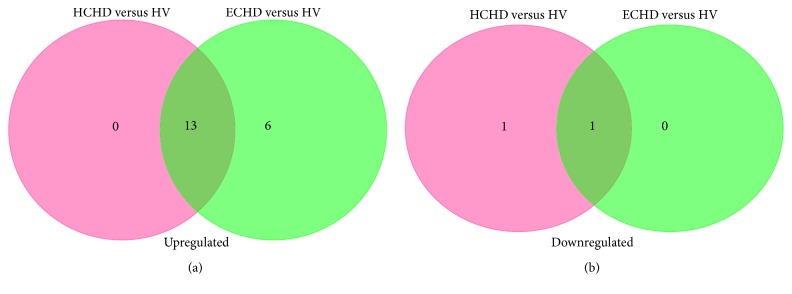
Venn diagrams of differentially upregulated microRNAs (a) and differentially downregulated miRNAs (b) in HCHD and ECHD patients.

MiRNAs from HCHD and ECHD patients were identified by microarrays. Compared with the healthy patients, 15 miRNAs with significantly differential expression (FDR adjusted *p* value < 0.05 and fold change > 1.5) were identified in HCHD group (Table 1). Among them, 13 miRNAs were overexpressed and 2 were underexpressed. Twenty differentially expressed miRNAs were detected in ECHD group relative to HV, namely, 19 upregulated miRNAs and 1 downregulated miRNA (Table 2). Unsupervised hierarchic clustering was carried out based on the differentially expressed miRNAs and displayed as heat maps (Figure 1). The differentially expressed miRNAs overlapping and nonoverlapping between HCHD and ECHD groups were presented in Figure 2. Thirteen differentially expressed miRNAs that were upregulated were detected in both HCHD and ECHD patients (Figure 2(a)). One downregulated miRNA was significantly differentially expressed between ECHD and HV groups. And the downregulated miRNA was also differentially expressed miRNA in HCHD group (Figure 2(b)).

### 3.2. Gene Ontology (GO) Analysis

**Figure 3:**
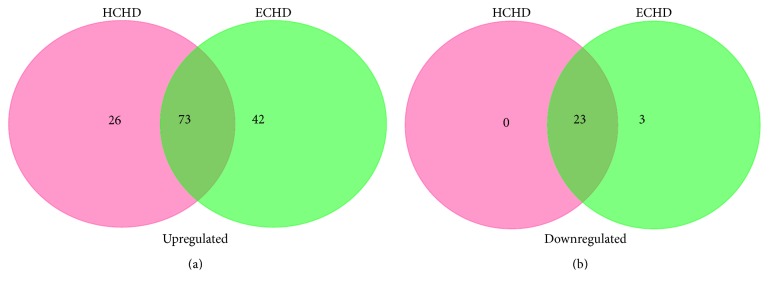
Venn diagrams of upregulated functional annotations (a) and downregulated functional annotations (b) from the target genes of differentially expressed miRNAs in HCHD and ECHD patients.

**Figure 4:**
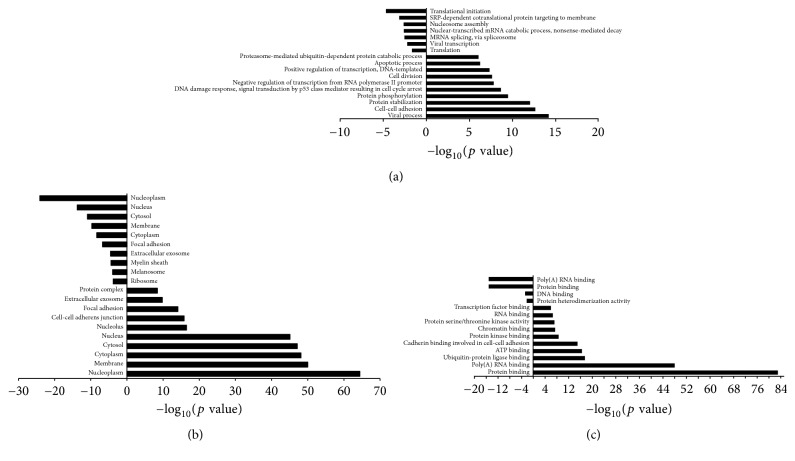
Significant upregulated and downregulated functional annotations of differentially expressed miRNAs in HCHD group. GO analysis according to biological process (a), cellular component (b), and molecular function (c), respectively, ranked by enrichment score (−log_10_(*p* value)).

**Figure 5:**
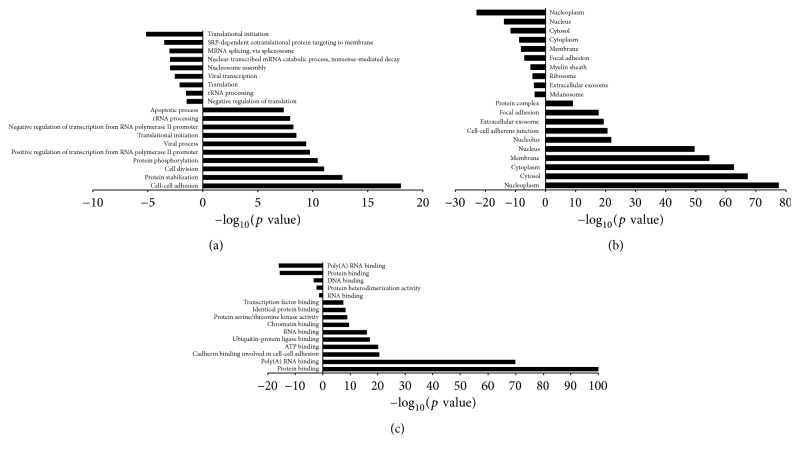
Significant upregulated and downregulated functional annotations of differentially expressed miRNAs in HCHD group. GO analysis according to biological process (a), cellular component (b), and molecular function (c), respectively, ranked by enrichment score (−log_10_(*p* value)).

Gene ontology of miRNA targets was analyzed based on biological process, cellular component, and molecular function. The overlapping and nonoverlapping functional annotations between HCHD and ECHD groups were shown in Venn diagrams (Figure 3). One hundred and twenty-two GO terms and 141 GO terms were significantly regulated (FDR < 0.05) by the differentially expressed miRNAs in HCHD group and ECHD group, respectively. Among them, 73 upregulated (Figure 3(a)) and 23 downregulated (Figure 3(b)) functional annotations targeted by the differentially expressed miRNAs were indicated in both HCHD and ECHD groups. Top 10 significant upregulated and downregulated functional annotations of differentially expressed miRNAs in HCHD and ECHD groups were presented in Figures 4 and 5. In HCHD group, the highly enriched GO terms in biological process (Figure 4(a)), cellular component (Figure 4(b)), and molecular function (Figure 4(c)) included viral process, protein stabilization, membrane, cytoplasm, ubiquitin-protein ligase binding, and ATP binding. As shown in Figure 5, the significant upregulated and downregulated functional annotations of differentially expressed miRNAs in ECHD group were cell-cell adhesion, translational initiation, nucleoplasm, nucleus, protein binding, and poly(A) RNA binding.

### 3.3. Pathway Analysis

**Figure 6:**
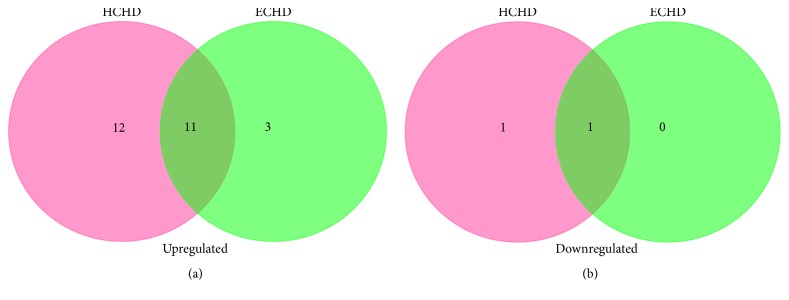
Venn diagrams of upregulated pathways (a) and downregulated pathways (b) from the target genes of differentially expressed miRNAs in HCHD and ECHD patients.

The pathways significantly (FDR < 0.05) affected by differentially expressed miRNAs were identified using KEGG pathway analysis. Eleven upregulated pathways (Figure 6(a)) that included cell cycle, viral carcinogenesis, chronic myeloid leukemia, and p53 signaling pathway were identified in both HCHD and ECHD groups. And 1 downregulated overlapping pathway (Figure 6(b)) was shared by both HCHD and ECHD groups. Furthermore, 12 upregulated pathways and 1 downregulated pathway, including hippo signaling pathway, signaling pathways regulating pluripotency of stem cells, PI3K-Akt signaling pathway, and TGF-beta signaling pathway, were only identified in HCHD group. And the top 10 significantly upregulated pathways and one significantly downregulated pathway were shown in Table 3. Three specific pathways were potentially affected by the differentially expressed miRNAs in ECHD group. The significantly upregulated pathways were presented in Table 4.

### 3.4. miRNA-Gene Regulatory Network Analysis

**Figure 7:**
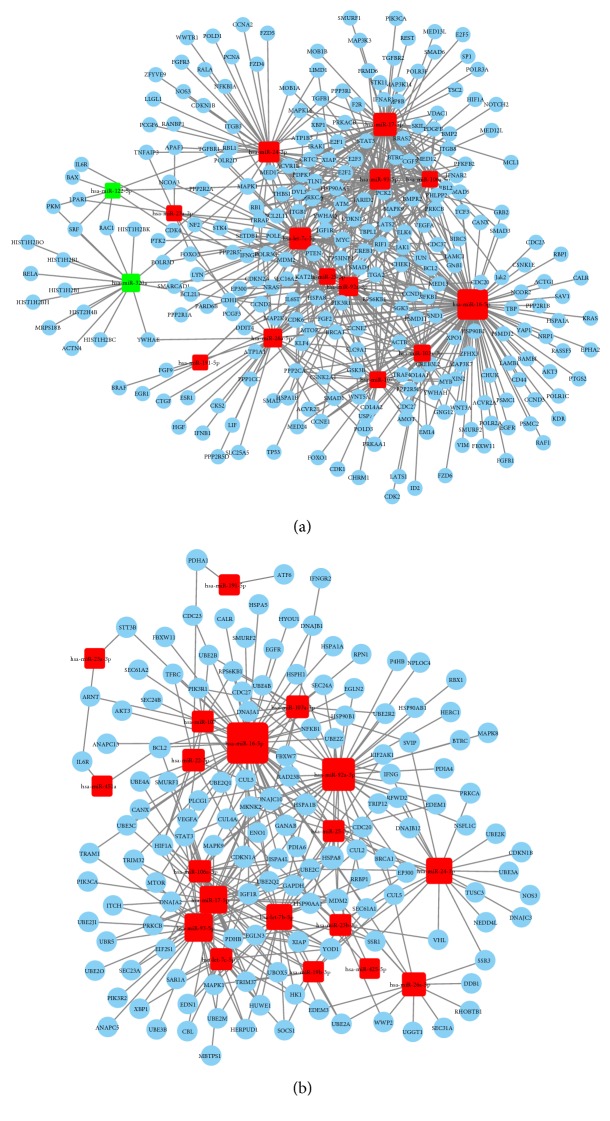
MiRNA-gene regulatory network diagram of HCHD (a) and ECHD (b). Red squares represent upregulated miRNAs and green squares represent downregulated miRNAs. Blue circles represent target genes. Black lines represent the regulatory relation between miRNAs and their target genes.

The miRNA-gene regulatory networks based on the genes that significantly regulated pathways were used to identify the putative target genes of the upregulated and downregulated miRNAs, which was shown in Figure 7. The betweenness centrality was used for topological analysis and the circles represented target genes and the squares represented miRNAs in the networks. In HCHD group, the total number of genes and miRNAs in the network was 272 and 15, respectively (Figure 7(a)). And the network diagram suggested the core miRNAs, namely, hsa-miR-16-5p, hsa-miR-17-5p, hsa-let-7c-5p, and hsa-miR-24-3p, and their targets. Four dysregulated miRNAs, namely, hsa-miR-16-5p, hsa-miR-92a-3p, hsa-miR-93-5p, and hsa-miR-17-5p, were core miRNAs in EHCD group (Figure 7(b)).

## 4. Discussion

The present study revealed differentially expressed circulating miRNAs between healthy volunteers and HCHD patients or ECHD patients. MiRNAs play an integral part in identifying the occurrence and development of various diseases and may be used as a new form of potential biomarkers [22]. Indeed, they are absolutely vital for the development of tissue and relevant to the pathological processes of numerous cardiovascular diseases, including acute myocardial infarction, heart failure, coronary artery disease, stroke, and hypertension [23]. MiRNA expression in HCHD and ECHD patients was explored by miRNA microarray analysis. According to the analysis, 1 specific miRNA was found in HCHD patients. Results indicated that hsa-miR-122-5p might be in connection with hyperglycemia in CHD patients. In the meantime, 6 specific miRNAs, including hsa-miR-451a, hsa-miR-425-5p, and hsa-let-7b-5p, were only found in EHCD patients. Thirteen significantly upregulated miRNAs and 1 significantly downregulated miRNA are overlapped in the HCHD and ECHD groups; the most significant upregulated and downregulated miRNAs are hsa-miR-16-5p and hsa-miR-320a, respectively. This suggested that hsa-miR-16-5p and hsa-miR-320a might be closely connected to the occurrence of CHD. MiR-16 is related to cardiovascular diseases. Because miR-16 can downregulate expressions of VEGFR2 and FGFR1 to inhibit angiogenesis, its expression change also can induce myocardial cell hypertrophy [24]. MiR-16 has been reported to play a key role in regulation of insulin signaling through a reduction in TNF*α* and suppressor of cytokine signaling 3 (SOCS3) signaling and increase in insulin-like growth factor binding protein-3 (IGFBP-3) levels to inhibit insulin resistance [25].

Target prediction by 2 databases (Tarbase and miRTarBase) identified large quantities of putative target genes for the differentially expressed miRNAs of HCHD and ECHD patients. And functional annotation by DAVID disclosed the biological properties and molecular functions of these target genes. The gene ontologies of HCHD and ECHD groups were compared to better understand the difference of biological property and molecular function of circulating miRNA profiles between HCHD and ECHD patients. The significantly upregulated and downregulated functional annotations of the miRNA targets in HCHD group included transcription, DNA-templated, protein polyubiquitination, regulation of cellular response to heat, nuclear membrane, nuclear pore, RISC complex, ubiquitin-protein ligase activity, ubiquitin-protein transferase activity, and double-stranded RNA binding, which suggested that hyperglycemia in CHD patients might relate to the exceptions of above biological processes and molecular functions.

In KEGG pathway analysis, a total of 25 significantly enriched pathways were found in HCHD group and a total of 15 significantly enriched pathways were found in ECHD group. TNF signaling pathway as the most significant upregulated pathway was found in HCHD group. It has been reported that TNF was shown to inhibit insulin signaling by serine phosphorylation of insulin receptors and protein phosphate PP-1 and by activation of protein tyrosine phosphatase SH-PTPase [26]. Therefore, it was speculated that the activation of TNF signaling pathway might be a reason for the occurrence of hyperglycemia in CHD patients. In addition, Hippo signaling pathway, signaling pathways regulating pluripotency of stem cells, PI3K-Akt signaling pathway, and TGF-beta signaling pathway were the top 10 significant upregulated pathways in HCHD group. PI3K/Akt signaling pathway is closely linked to plenty of biological processes such as proliferation, survival, migration, and cellular metabolic regulation and it is responsible for endothelial dysfunction and cell apoptosis caused by hyperglycemia [27]. PI3K/Akt signaling pathway which is thought to play an important role in insulin's metabolic function is a primary way to regulate the absorption and metabolism of glucose and balance the glucose level in blood [28]. Some studies have shown that insulin regulates blood glucose primarily through PI3K/Akt signaling pathway [29]. Wnt signaling pathway was found in ECHD group as a significant upregulated pathway. Wnt signaling pathway seems to be playing an important role in the occurrence of hypertension and diabetes, which has been demonstrated by clinical and in vitro studies [30]. Other studies exhibited an association between Wnt signaling pathway and the regulation of insulin secretion in *β*-cell [31]. It has been reported that activated Wnt signaling increases insulin secretion in isolated mouse islets and attenuated Wnt signaling decreases insulin content in both isolated mouse and human islets [32]. Wnt signaling pathway was significant upregulated pathway in ECHD group. Therefore, the activation of Wnt signaling pathway might be the main reason that patients with CHD kept their blood glucose in the normal levels.

MiRNA-gene regulatory networks were constructed to investigate the function of differentially expressed miRNAs in HCHD and ECHD groups. A detailed relationship between differentially expressed miRNAs and genes was found in HCHD groups. Results illustrated that upregulated miRNAs might play a more important role in the regulation of HCHD. Because hsa-miR-16-5p and hsa-miR-17-5p were core miRNAs in both HCHD and ECHD groups, these 2 miRNAs might play a crucial role in the occurrence and development of CHD. In addition, hsa-let-7c-5p and hsa-miR-24-3p might have the most important function for hyperglycemia in CHD patients and hsa-miR-92a-3p and hsa-miR-93-5p also were crucial in ECHD patients.

## 5. Conclusions

In the present study, we investigated the common and different mechanisms of miRNAs in HCHD and ECHD patients by comparing the biological properties, molecular functions, and enriched pathways of target genes of their differentially expressed circulating miRNAs. The biological characteristics of HCHD and ECHD and the molecular alterations in these two types of CHD were better understood through the study, which is conducive to explore novel therapies for future clinical applications to improve therapeutic efficacy and pertinence of treatment.

## Figures and Tables

**Table 1:** Thirteen overexpressed and 2 underexpressed circulating miRNAs of HCHD patients.

Systematic name	FDR adjusted *p* value	Fold change	Regulation
hsa-miR-24-3p	0.0257	1.5438	Up
hsa-miR-26a-5p	0.0257	1.5757	Up
hsa-let-7c-5p	0.0211	1.7598	Up
hsa-miR-107	0.0333	1.8457	Up
hsa-miR-92a-3p	0.0292	1.8829	Up
hsa-miR-103a-3p	0.0331	1.9849	Up
hsa-miR-25-3p	0.0257	2.0408	Up
hsa-miR-93-5p	0.0302	2.1112	Up
hsa-miR-106a-5p	0.0331	2.1919	Up
hsa-miR-191-5p	0.0454	2.2617	Up
hsa-miR-17-5p	0.0211	2.3144	Up
hsa-miR-16-5p	0.0211	2.3306	Up
hsa-miR-23a-3p	0.0257	2.4398	Up
hsa-miR-122-5p	0.0412	1.5832	Down
hsa-miR-320a	0.0467	2.2958	Down

**Table 2:** Nineteen overexpressed miRNAs and 1 underexpressed circulating miRNA of ECHD patients.

Systematic name	FDR adjusted *p* value	Fold change	Regulation
hsa-miR-23b-3p	0.0329	1.5214	Up
hsa-miR-19b-3p	0.0235	1.5383	Up
hsa-miR-26a-5p	0.0304	1.5392	Up
hsa-miR-22-3p	0.0304	1.6269	Up
hsa-let-7b-5p	0.0101	1.6539	Up
hsa-miR-24-3p	0.0304	2.0320	Up
hsa-miR-103a-3p	0.0327	2.1242	Up
hsa-miR-107	0.0327	2.1653	Up
hsa-miR-92a-3p	0.0326	2.1859	Up
hsa-miR-451a	0.0479	2.2234	Up
hsa-miR-25-3p	0.0304	2.3831	Up
hsa-let-7c-5p	0.0104	2.5818	Up
hsa-miR-17-5p	0.0205	2.5950	Up
hsa-miR-93-5p	0.0327	2.6229	Up
hsa-miR-191-5p	0.0393	2.6341	Up
hsa-miR-106a-5p	0.0327	2.6382	Up
hsa-miR-425-5p	0.0473	2.7734	Up
hsa-miR-23a-3p	0.0304	2.8259	Up
hsa-miR-16-5p	0.0205	3.0602	Up
hsa-miR-320a	0.0409	1.9496	Down

**(a):** 

KEGG pathway	FDR adjusted *p* value	Genes	miRNAs
TNF signaling pathway	4.8*E*–04	30	12
Hippo signaling pathway	0.0013	52	14
Signaling pathways regulating pluripotency of stem cells	0.0017	49	12
Non-small cell lung cancer	0.003	26	12
Epstein-Barr virus infection	0.004	60	12
HTLV-I infection	0.010	74	14
Small cell lung cancer	0.011	33	13
PI3K-Akt signaling pathway	0.015	93	14
Glioma	0.022	27	13
TGF-beta signaling pathway	0.024	32	11

**(b):** 

KEGG pathway	FDR adjusted *p* value	Genes	miRNAs
Viral carcinogenesis	0.019	20	2

**Table 4:** Specific pathways enriched by targets of differentially expressed miRNAs in ECHD group.

KEGG pathway	FDR adjusted *p* value	Genes	miRNAs
Ubiquitin mediated proteolysis	0.005	50	15
Wnt signaling pathway	0.019	38	16
Protein processing in endoplasmic reticulum	0.033	56	19
